# Correlating Molecular Phylogeny with Venom Apparatus Occurrence in Panamic Auger Snails (Terebridae)

**DOI:** 10.1371/journal.pone.0007667

**Published:** 2009-11-05

**Authors:** Mandë Holford, Nicolas Puillandre, Maria Vittoria Modica, Maren Watkins, Rachel Collin, Eldredge Bermingham, Baldomero M. Olivera

**Affiliations:** 1 The City University of New York-York College & CUNY Graduate Center and The American Museum of Natural History, New York, New York, United States of America; 2 Department of Biology, University of Utah, Salt Lake City, Utah, United States of America; 3 Département Systématique et Évolution, Muséum National d'Histoire Naturelle, Paris, France; 4 Dipartimento di Biologia Animale e dell'Uomo, Università di Roma “La Sapienza,” Rome, Italy; 5 Smithsonian Tropical Research Institute, Panama City, Panama; Northeastern University, United States of America

## Abstract

Central to the discovery of neuroactive compounds produced by predatory marine snails of the superfamily Conoidea (cone snails, terebrids, and turrids) is identifying those species with a venom apparatus. Previous analyses of western Pacific terebrid specimens has shown that some Terebridae groups have secondarily lost their venom apparatus. In order to efficiently characterize terebrid toxins, it is essential to devise a key for identifying which species have a venom apparatus. The findings presented here integrate molecular phylogeny and the evolution of character traits to infer the presence or absence of the venom apparatus in the Terebridae. Using a combined dataset of 156 western and 33 eastern Pacific terebrid samples, a phylogenetic tree was constructed based on analyses of 16S, COI and 12S mitochondrial genes. The 33 eastern Pacific specimens analyzed represent four different species: *Acus strigatus, Terebra argyosia, T. ornata*, and *T*. cf. *formosa*. Anatomical analysis was congruent with molecular characters, confirming that species included in the clade *Acus* do not have a venom apparatus, while those in the clade *Terebra* do. Discovery of the association between terebrid molecular phylogeny and the occurrence of a venom apparatus provides a useful tool for effectively identifying the terebrid lineages that may be investigated for novel pharmacological active neurotoxins, enhancing conservation of this important resource, while providing supplementary information towards understanding terebrid evolutionary diversification.

## Introduction

The auger snails (family Terebridae) are a distinctive group of carnivorous, sand-dwelling gastropods included in the superfamily Conoidea, along with cone snails and turrids [1]. Species in this large gastropod superfamily (>10,000 species) generally use venom to capture their prey [2], [3]. Conoidean venoms are of considerable interest as they are a rich source of neuroactive peptides, widely used to investigate cellular communication in the nervous system [4]–[6]. Some Conoidean venom components have been used directly for a variety of biomedical applications [7], [8]. Several peptides from cone snail venoms have reached human clinical trials, and one (Prialt) has been approved as a drug for intractable pain [9], [10].

In contrast to cone snail toxins (conotoxins), terebrid toxins are largely uncharacterized and no physiological target for any terebrid venom peptide has been defined. However, the very preliminary characterization carried out to date suggests that the venoms of the Terebridae have novel components, distinct from other conoidean venoms [11], [12]. Thus, terebrid venoms are potentially a rich, unexplored pharmacological resource.

A significant fraction of the ∼300–400 species in the Terebridae do not have the characteristic anatomical structures that comprise the venom delivery apparatus of conoidean snails, namely a venom bulb, venom duct, and radula sac [13]–[16]. Analysis of shell morphology alone cannot generally determine whether or not a species in the Terebridae has a venom apparatus. The non-monophyly of most of the terebrid genera makes the attribution of a specimen to a particular genus challenging. Therefore, identifying *a priori* which species to collect for the analysis of venom components is problematic.

The first molecular phylogeny of the Terebridae based on a three-gene matrix of molecular markers 12S, 16S, and cytochrome oxidase I (COI), was recently published[16]. The data suggest that the family Terebridae could be divided into at least 5 distinctive generic clades: *Acus, Terebra, Hastula, Myurella*, and a sister clade of the four others that includes *Terebra jungi* (recently revised to *Pellifronia jungi* [17]). Furthermore, based on species clusters, it was suggested that molecular data may be a useful tool to identify which terebrid species have a venom apparatus and which do not. For these molecular criteria to reliably indicate which species of terebrids are venomous, the criteria should be applicable to all Terebridae.

The original correlation between venom apparatus and molecular phylogeny was established using only western Pacific species [16]. This paper examines the validity of correlating molecular phylogeny and venom apparatus by increasing the diversity of taxa sampled and the geographic coverage to include terebrid samples from the eastern Pacific. There are currently 55 described species of terebrids found in the Panamic fauna as defined by Keen [18]. In terms of geographic distribution, the Panamic tropical molluscan marine fauna is arguably highly divergent from that of the western Pacific. Thus, whether the molecular phylogeny/venom apparatus correlation established for western Pacific terebrid samples can be used to assess eastern Pacific terebrid snails is a central issue addressed by this study. Presented here is the first molecular analysis of Panamic Terebridae, which is used to highlight both phylogenetic and taxonomic issues for this group.

## Materials and Methods

### Material

**Figure 1:**
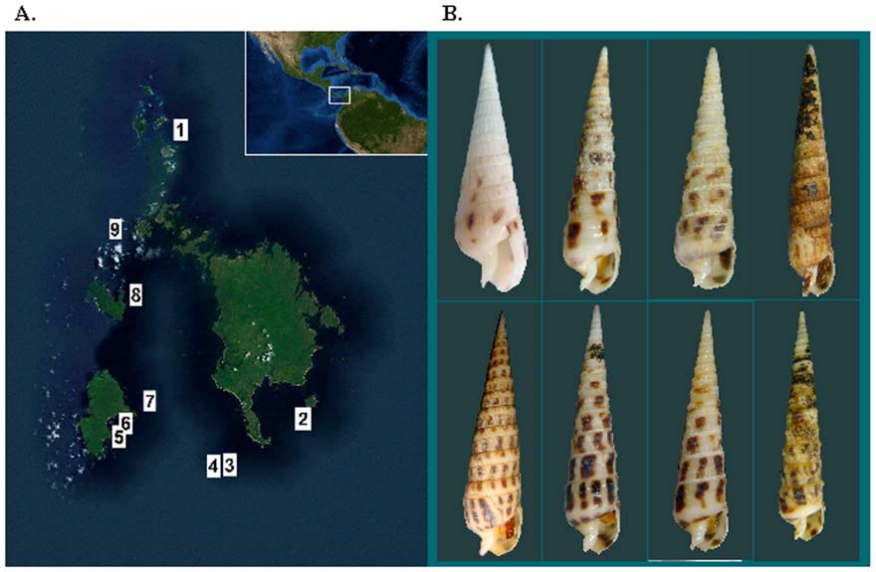
Panamic terebrid collection site and specimens. A. The Las Perlas Archipelago, located off the west coast of Panama (see Inset), is the collection site for the terebrids analyzed. The numbers shown on the map refer to the stations for the Panamic specimens listed in Table 1. B. Las Perlas specimens of *Acus* and *Terebra* analyzed in this study. Top left, *Acus strigatus*. Bottom left, *Terebra ornata*. Top right-most specimen, *Terebra* cf. *formosa*. All other specimens are *Terebra argyosia*.

Panamic specimens used were dredged from the Las Perlas Archipelago in 2008, using The Smithsonian Tropical Research Institute research vessel RV-Urraca. The collected material was specifically fixed for molecular and anatomical analysis. Living specimens were anesthetized in MgCl_2_ isotonic with seawater for 1 or 2 hours. Samples were dissected and a piece of tissue (usually foot) was fixed in 95% ethanol. Table 1 lists all terebrid specimens used in this study, including the specific geographical coordinates of where they were collected (for map, see Figure 1). Taxonomic assignments were made based on shell morphology. Vouchers of the Panamic specimens are deposited in the Muséum National d'Histoire Naturelle (MNHN) of Paris. Included with the 33 Panamic taxa are sequences from specimens collected in the western Pacific and analyzed in Holford et al. 2009 [16]. Outgroups are identical to those used in Holford et al. 2009 [16] and identified in Table 1.

**Table 1:** List of terebrid samples used in this study. VA =  venom apparatus.

*Genus*	*Species*	COI	12S	16S	VA	Station number - Coordinates/Depth	MNHNnumber
**Panamic Specimens**							
*Acus*	*strigatus (Sowerby, 1825)*	x	x	x	No	3–08°11.8′N, 078°57.1′W/21.4 m	42093
*Acus*	*strigatus (Sowerby, 1825)*	x	x	x	No	4–08°11.8′N, 078°57.5′W/22.4 m	42105
*Acus*	*strigatus (Sowerby, 1825)*	x	x	x	No	5–08°14.7′N, 079°05.55′W/17.5 m	42136
*Acus*	*strigatus (Sowerby, 1825)*	x	x	x	No	5–08°14.7′N, 079°05.55′W/17.5 m	42137
*Acus*	*strigatus (Sowerby, 1825)*		x	x	No	9–08°30.1′N, 079°06.0′W/21 m	42159
*Terebra*	*argosyia (Olsson, 1971)*	x	x	x	Yes	1–08°37.18′N, 079°01.12′W/25 m	42068
*Terebra*	*argosyia (Olsson, 1971)*	x	x	x	Yes	1–08°37.18′N, 079°01.12′W/25 m	42069
*Terebra*	*argosyia (Olsson, 1971)*			x	Yes	2–08°15.61′N, 078°51.57′W/24.1 m	42071
*Terebra*	*argosyia (Olsson, 1971)*	x	x	x	Yes	2–08°15.61′N, 078°51.57′W/24.1 m	42072
*Terebra*	*argosyia (Olsson, 1971)*	x	x	x	Yes	2–08°15.61′N, 078°51.57′W/24.1 m	42073
*Terebra*	*ornata (Gray, 1834)*			x	Yes	1–08°37.18′N, 079°01.12′W/20 m	42074
*Terebra*	*argosyia (Olsson, 1971)*	x	x	x	Yes	3–08°11.8′N, 078°57.1′W/21.4 m	42084
*Terebra*	*argosyia (Olsson, 1971)*	x	x	x	Yes	3–08°11.8′N, 078°57.1′W/21.4 m	42085
*Terebra*	*argosyia (Olsson, 1971)*	x	x	x	Yes	3–08°11.8′N, 078°57.1′W/21.4 m	42086
*Terebra*	*argosyia (Olsson, 1971)*	x	x	x	Yes	3–08°11.8′N, 078°57.1′W/21.4 m	42087
*Terebra*	*argosyia (Olsson, 1971)*	x	x	x	Yes	3–08°11.8′N, 078°57.1′W/21.4 m	42089
*Terebra*	*argosyia (Olsson, 1971)*	x	x	x	Yes	3–08°11.8′N, 078°57.1′W/21.4 m	42090
*Terebra*	*argosyia (Olsson, 1971)*	x	x	x	Yes	3–08°11.8′N, 078°57.1′W/21.4 m	42091
*Terebra*	*argosyia (Olsson, 1971)*	x	x	x	Yes	3–08°11.8′N, 078°57.1′W/21.4 m	42092
*Terebra*	*argosyia (Olsson, 1971)*	x	x	x	Yes	4–08°11.8′N, 078°57.5′W/24 m	42099
*Terebra*	*argosyia (Olsson, 1971)*	x	x	x	Yes	4–08°11.8′N, 078°57.5′W/24 m	42100
*Terebra*	*argosyia (Olsson, 1971)*			x	Yes	4–08°11.8′N, 078°57.5′W/24 m	42102
*Terebra*	*argosyia (Olsson, 1971)*	x	x	x	Yes	4–08°11.8′N, 078°57.5′W/22.4 m	42103
*Terebra*	*argosyia (Olsson, 1971)*	x	x	x	Yes	4–08°11.8′N, 078°57.5′W/22.4 m	42104
*Terebra*	*argosyia (Olsson, 1971)*	x	x	x	Yes	4–08°11.8′N, 078°57.5′W/22.4 m	42118
*Terebra*	*argosyia (Olsson, 1971)*	x	x	x	Yes	4–08°11.8′N, 078°57.5′W/22.4 m	42119
*Terebra*	*argosyia (Olsson, 1971)*	x	x	x	Yes	4–08°11.8′N, 078°57.5′W/22.4 m	42120
*Terebra*	*argosyia (Olsson, 1971)*	x	x	x	Yes	4–08°11.8′N, 078°57.5′W/22.4 m	42121
*Terebra*	*argosyia (Olsson, 1971)*	x	x	x	Yes	4–08°11.8′N, 078°57.5′W/22.4 m	42122
*Terebra*	*argosyia (Olsson, 1971)*	x	x	x	Yes	4–08°11.8′N, 078°57.5′W/22.4 m	42123
*Terebra*	*argosyia (Olsson, 1971)*	x	x	x	Yes	4–08°11.8′N, 078°57.5′W/22.4 m	42124
*Terebra*	*argosyia (Olsson, 1971)*	x	x	x	Yes	4–08°11.8′N, 078°57.5′W/22.4 m	42125
*Terebra*	*ornata (Gray, 1834)*	x	x	x	Yes	6–08°14.94′N, 079°05.7′W/14.3 m	42131
*Terebra*	*cf. formosa*	x	x	x	Yes	7–08°16.86′N, 079°02.67′W/39.2 m	42152
*Terebra*	*argosyia (Olsson, 1971)*		x	x	Yes	8–08°24.50′N, 079°04.66′W/18.4 m	42153
**IndoPacific Specimens**							
*Acus*	*maculatus (Linnaeus, 1758)*	x	x	x	No	9°37.4′N, 123°46.9′E, 3–20 m	30370
*Acus*	*dimidiatus (Linnaeus, 1758)*	x	x	x	No	15°32.5′S, 167°10.5′E, 5–10 m	30372
*Acus*	*dimidiatus (Linnaeus, 1758)*	x	x	x	No	15°36.9′S, 167°10.5′E, 6–33 m	30373
*Acus*	*crenulatus (Linnaeus, 1758)*	x	x	x	No	15°34.4′S, 167°13.1′E, 9 m	30377
*Acus*	*dimidiatus (Linnaeus, 1758)*	x	x	x	No	15°32.5′S, 167°10.5′E, 5–10 m	30379
*Acus*	*dimidiatus (Linnaeus, 1758)*	x	x	x	No	15°35.4′S, 166°59.7′E, 3–37 m	30381
*Acus*	*maculatus (Linnaeus, 1758)*	x	x	x	No	15°28.7′S, 167°15.2′E, 19 m	30389
*Acus*	*dimidiatus (Linnaeus, 1758)*	x	x	x	No	15°38.1′S, 167°05.9′E, intertidal	30428
*Acus*	*felinus (Dillwyn, 1817)*	x	x	x	No	9°37.4′N, 123°54.5E, 6–8 m	30443
*Acus*	*felinus (Dillwyn, 1817)*	x	x	x	No	9°37.4′N, 123°54.5E, 6–8 m	30445
*Acus*	*chloratus (Lamarck. 1822)*	x	x	x	No	15°22.6′S, 167°11.6′E, intertidal	30490
*Acus*	*crenulatus (Linnaeus, 1758)*	x	x	x	No	15°34.4′S, 167°13.1′E, 9 m	30494
*Acus*	*areolatus (Link, 1807)*	x	x	x	No	9°37.4′N, 123°46.9′E, 3–20 m	30587
*Cinguloterebra*	*cf. fujitai (Kuroda & Habe, 1952)*	x	x	x	Yes	9°27.4′N, 123°49.4′E, 273–356 m	15724
*Cinguloterebra*	*cf. fenestrata (Hinds, 1844)*	x	x	x	Yes	9°36.2′N, 123°43.8′E, 382–434 m	16735
*Cinguloterebra*	*cf. fenestrata (Hinds, 1844)*	x	x	x	Yes	9°29.4′N, 123°44.4′E, 271–318 m	30390
*Cinguloterebra*	*triseriata (JE Gray, 1824)*	x	x	x	Yes	9°35.3′N, 123°52.2′E, 84–87 m	30404
*Cinguloterebra*	*fenestrata type I*	x	x	x	Yes	9°39.2′N, 123°47.5′E, 255–268 m	30410
*Cinguloterebra*	*fenestrata type II*	x	x	x	Yes	9°39.2′N, 123°47.5′E, 255–268 m	30418
*Cinguloterebra*	*lima (Deshayes, 1857)*	x	x	x	Yes	15°32.5′S, 167°10.5′E, 5–10 m	30485
*Cinguloterebra*	*lima (Deshayes, 1857)*	x	x	x	Yes	8°39.5′ S, 157°23.0′ E, 214–243 m	30487
*Cinguloterebra*	*jenningsi (RD Burch. 1965)*	x	x	x	Yes	15°28.6′S, 167°15.1′E, 3–31 m	30544
*Cinguloterebra*	*anilis (Röding, 1798)*	x	x	x	Yes	15°35.2′S, 167°59.4′E, intertidal	30552
*Hastula*	*strigilata (Linnaeus, 1758)*	x	x	x	Yes	15°35.2′S, 167°59.4′E, intertidal	30420
*Myurella*	*affinis (JE Gray 1834)*	x	x	x	No	9°37.4′N, 123°54.5′E, 6–8 m	30430
*Terebra*	*guttata (Röding, 1798)*	x	x	x	Yes	15°33.1′S, 167°12.2′E, 3–40 m	30376
*Terebra*	*babylonia (Lamarck. 1822)*	x	x	x	Yes	15°31.1′S, 167°10.5′E, 7 m	30380
*Terebra*	*subulata (Linnaeus, 1767)*	x	x	x	Yes	15°36.6′S, 167°10.1′E, 8–20 m	30386
*Terebra*	*guttata (Röding, 1798)*	x	x	x	Yes	15°33.1′S, 167°12.2′E, 3–40 m	30387
*Terebra*	*laevigata (JE Gray, 1834)*	x	x	x	Yes	15°36.9′S, 167°10.5′E, 6–33 m	30394
*Terebra*	*tricolor(GB Sowerby I, 1825)*	x	x	x	Yes	15°33.1′S, 167°17.8′E, 15–25 m	30409
*Terebra*	*laevigata (JE Gray, 1834)*	x	x	x	Yes	9°36.8′N, 123°52.2′E, intertidal	30431
*Terebra*	*subulata (Linnaeus, 1767)*	x	x	x	Yes	9°37.4′N, 123°54.5E, 6–8 m	30444
*Terebra*	*subulata (Linnaeus, 1767)*	x	x	x	Yes	9°32.8′N, 123°42.1′E, 3–35 m	30483
*Terebra*	*tricolor(GB Sowerby I, 1825)*	x	x	x	Yes	15°38.5′S, 167°15.1′E, 13 m	30493
*Terebra*	*laevigata (JE Gray, 1834)*	x	x	x	Yes	15°26.6′S, 167°15.2′E, intertidal	30597
*Terebra*	*laevigata (JE Gray, 1834)*	x	x	x	Yes	15°43.4′S, 167°15.0′E, 6 m	30603
*Terebra*	*laevigata (JE Gray, 1834)*	x	x	x	Yes	15°31′S, 167°09′E, intertidal	30613
*Terebra*	*laevigata (JE Gray, 1834)*	x	x	x	Yes	15°31′S, 167°09′E, intertidal	30632
*Pellifronia*	*jungi (Lai, 2001)*	x	x	x	Yes	9°37.5′N, 123°40.2′E, 606–631 m	30395
**Outgroups**							
*Cochlespira sp. (Turridae)*		x	x	x		21°10′S, 158°39′E, 650–723 m	40568
*Conus nereis (Conidae)*		x	x	x	Yes	9°32.5′N, 123°41.8′E, 111–115 m	17922
*Harpa sp. (Harpidae)*		x	x	x		9°32.5′N, 123°41.8′E, 111–115 m	40569
*Iotyrris cingulifera (Turridae)*		x	x	x		15°33.6′S, 167°16.6′E, 8–9 m	17685

### Sequencing

DNA was extracted from foot or other tissue using Qiagen QIAamp Dneasy Tissue kit. Fragments of mitochondrial genes 12S, 16S and COI were amplified using universal primers 12S1/12S3 [19], 16Sar/16Sbr [20], and LCO1490/HCO2198 [21] respectively. PCR reactions were performed in 25 µl, containing 3 ng of DNA, 10X reaction buffer, 2.5 mM MgCl_2_, 0.26 mM dNTP, 0.3 mM each primer, 5% DMSO, and 1.5 units of Qbiogene Q-Bio Taq or Advantage® 2 PCR Kit from Clontech. Amplification was performed as previously described [16]. PCR products were purified using USB ExoSAP-IT® or Quiagen PCR purification kit and sequenced. All genes were sequenced in both directions. Sequences were deposited in GenBank (Genbank accession numbers: FJ707376-FJ707472). Specimens data and COI sequences were also deposited in BOLD (Barcode of Life Data Systems, project CONO - Conoidea barcodes and taxonomy).

### Molecular and Phylogenetic analyses

COI sequences were manually aligned and 12S and 16S were automatically aligned using ClustalW multiple alignment implemented in BioEdit version 7.0.5.3 [22]. The accuracy of automatic alignments was confirmed by visual inspection. Hyper-variable regions of 12S and 16S genes were excluded from further analyses due to ambiguities in the alignments. All the western Pacific terebrid sequences obtained by Holford *et al.* 2009 [16] were included in this new dataset.

Phylogenetic analyses were based on reconstructions using two approaches: (i) Maximum Likelihood (ML) using PhyML 2.4.4 [23], where support of nodes were estimated with 100 bootstrap replicates, and (ii) Bayesian Analyses (BA) consisting of six Markov chains, 10,000,000 generations each, with a sampling frequency of one tree each thousand generations, run in four parallel analyses using MrBayes [24]. The number of swaps that are tried each time the chain stops for swapping was 4, and the chain temperature was set at 0.05. Twenty-five percent of the first generations were discarded as burnin, which correspond to the time the chain took to reach stationarity. For both ML and BA, the best-fitting model of evolution was applied, as determined by Modelgenerator V.85 following the Hierarchical Likelihood Ratio Test (with four discrete gamma categories). Variation was partitioned among genes and gene-specific model parameters were used. Each gene was first analysed separately and then the combined dataset was analysed. For the combined dataset one model of evolution for the concatenation of the three genes was used for the ML analysis. For the BA, a different model was applied for each gene as determined by Modelgenerator.

## Results

### Distribution of the Panamic Terebridae

The 33 Panamic specimens analyzed were assigned to four different terebrid species: *Acus strigatus, Terebra argyosia, T. ornata, and T.* cf. *formosa*. All taxonomic assignments made are based on shell morphology and later confirmed by molecular results. The *T. argyosia* specimens (collection sites 1, 2, 3, and 4) appear to be present both in the northern and southern ends of the archipelago (Figure 1A). *A. strigatus* was found between Punta Coco on Isla Del Rey and San Jose (sites 4 and 6). *T. ornata* was collected along the eastern coast of San Jose (site 5) and *Terebra* cf. *formosa* at site 7. Examples of the actual specimens analyzed are shown in Figure 1B.

### Phylogenetic analyses

**Figure 2:**
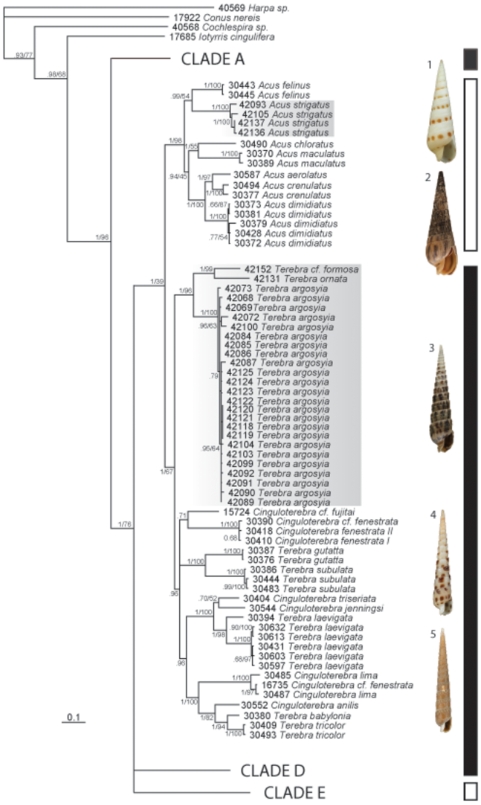
Combined Phylogenetic analysis of Panamic and western Pacific Terebridae. Shown is a consensus tree (BA) using COI, 16S, and 12S data sets. Posterior probabilities and bootstrap values are specified for each node. Shaded clades were collected in Panama. The bar on the right shows which taxa have venom glands (black bars) and which do not (white bars). Clade A refers to the sister group that includes *Pellifronia jungi*, Clades D and E refer to the *Hastula* and *Myurella* clades respectively; these clades were identified previously. Representative shells are shown as follows: 1. *Acus felinus*. 2. *Acus strigatus*. 3. *Terebra argosyia*. 4. *Terebra subulata*. 5. *Cinguloterebra anilis*.

After alignment, DNA fragments of 658, 534, and 455 bp were obtained for COI, 12S, and 16S genes, respectively. No contradictions were observed when independently constructed gene trees for COI, 12S, and 16S genes were analyzed (results not shown). These Panamic sequences were combined with sequences from western Pacific terebrid specimens to reconstruct the phylogeny illustrated in Figure 2. The best model of evolution for the COI, 12S and 16S and for the combined dataset is GTR+I+G (General Time Reversible model, with invariant sites and a gamma law parameter) for all genes, with I = 0.51 and α = 0.68 for COI, I = 0.6 and α = 0.62 for 12S, I = 0.34 and α = 0.32 for 16S and I = 0.41 and α = 0.4 for the combined dataset. Results obtained with Maximum Likelihood (ML) and Bayesian analyses (BA) are highly similar, however, the support values for ML were generally weaker.

Of the 5 distinct terebrid clades previously identified, Clade A (*P. jungi*), Clade B (*Acus*), Clade C (*Terebra*), Clade D (*Hastula*), and Clade E (*Myurella*), the Panamic sequences reported here fall into the *Acus* and *Terebra* clades. As a result, in order to reduce the size of the tree and to focus on the Panamic clades, only the *Acus* and *Terebra* clades are detailed in Figure 2. The other clades, represented by a single branch, are identical to those in Holford *et al.* 2009 [16].

The phylogenetic analysis strongly indicates that the Panamic *Acus strigatus* specimens in our sampling are within the *Acus* clade (Posterior Probablity (PP)  = 1; Bootstraps (B)  = 98). The *Acus* clade comprises a prevalence of western Pacific species (*A. felinus, A. chloratus, A. maculatus, A. areolatus, A. crenulatus, and A. dimidatus*). The monophyly of the Panamic specimens identified as belonging to the *Terebra* clade is well-supported (PP = 1; B = 96) within this group. As illustrated in the tree there are three distinct Panamic species present, *Terebra argyosia, Terebra ornata,* and *Terebra* cf. *formosa*.

### Character evolution

All Panamic specimens collected were dissected and the presence or absence of a venom apparatus was noted (Table 1). The presence/absence of a venom apparatus is a character trait that can be correlated with the molecular phylogeny of these specimens. The character evolution of the venom apparatus in the Terebridae was mapped previously for western Pacific specimens [16], indicating this group has lost the venom apparatus at least twice during its evolution. As indicated in Figure 2, the Panamic species placed in the *Acus* clade, *A. strigatus*, did not have a venom apparatus (highlighted with a white box). However, *T. ornata, T. argyosia*, and *T*. cf. *formosa*, all have a venom apparatus and fall within the genus *Terebra*, which contains other terebrid species identified as having a venom apparatus [13], [25] (highlighted by a black box).

## Discussion

Predatory marine snails of the superfamily Conoidea produce several neurotoxins in their venom that are used to capture and subdue prey [26]–[28]. The characteristic venom apparatus of conoideans is not present in a significant fraction of species in the family Terebridae. For this work, four Panamic species, *Acus strigatus, Terebra argyosia*, *Terebra ornata*, and *Terebra cf. formosa*, were analyzed using a combination of molecular phylogeny and character trait evolution based on the presence or absence of a venom apparatus (Figure 2). The molecular characters are completely congruent with anatomical data: all specimens without a venom apparatus are in the *Acus* clade, and all specimens with a venom apparatus are in the *Terebra* clade. Thus, DNA sequences can be used to infer if a terebrid species has a venom apparatus or not. This study confirms the correlation between phylogeny and the presence or absence of the venom apparatus previously established [16]. The present findings can be used to broaden the current knowledge of the Terebridae as it pertains to their taxonomy and the potential use of their toxins to characterize ion channels and receptors in the nervous system.

### Terebrid taxonomic considerations

The three Panamic species *T. argyiosa*, *T*. cf *formosa* and *T*. *ornata* form a well supported monophyletic branch (PP = 1; B = 96) within the clade that includes the type species of the genus *Terebra*, *T. subulata*. Therefore we provisionally treat all species in this clade as belonging to the genus *Terebra*. Subgeneric divisions may be feasible, but it seems best to defer the comprehensive taxonomic treatment of the genus *Terebra* until greater taxon sampling has been achieved.

**Figure 3:**
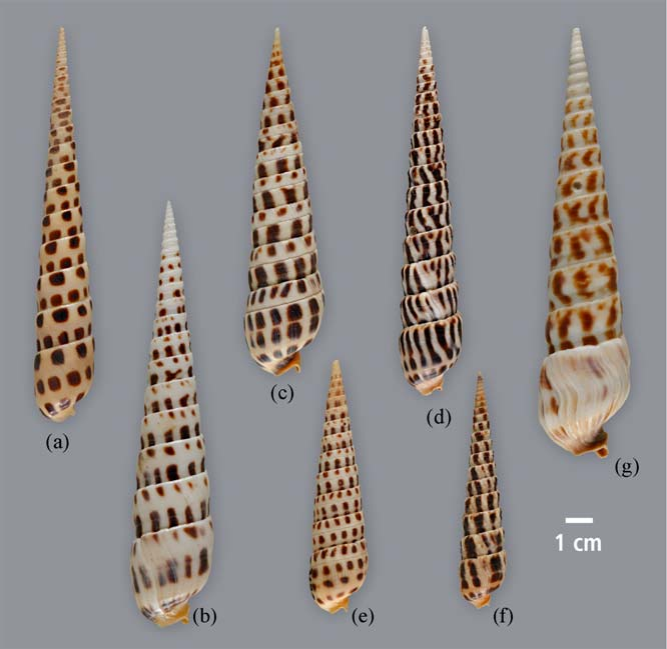
Diversity of Eastern Pacific *Terebra*. The figure shows the diversity of the venomous eastern Pacific forms tentatively assigned to Clade C, *Terebra*. The samples from Mexico, labeled (b–d), appear different to the samples from Panama, which are labeled (e–f). These are compared to the left-most specimen (a), *Terebra subulata* from the western Pacific and the right-most specimen (f), *Terebra taurina* from the western Atlantic.

The species-level taxonomy of *Terebra* species from the Panamic region is generally problematic. The results obtained so far provide a guide for suggesting which Panamic forms are likely to belong to *Terebra*, and thus have a venomous apparatus. However, considerable care should be taken before assigning definitive species designations for forms in this group. This problem is highlighted by the specimens of a variety of eastern Pacific terebrids shown in Figure 3. Note that the specimens assigned to *T. argyosia* and *T. ornata* from Mexico are quite different in shell pattern from the specimens from Panama. Two non-Panamic species are also included in the figure, a western Pacific species, *T. subulata*, and an Atlantic species that we expect will also belong to the same *Terebra* clade, *T. taurina*.

In this instance the molecular characters used in the phylogenetic analyses confirmed the shell-based morphological characters used to identify different terebrid species. The specimens of *Terebra argyosia* comprise the largest group of Las Perlas specimens collected that have a venom apparatus. Molecular analysis implies that *T. argyosia*, *T. ornata* and *T*. cf. *formosa* are indeed three different species. However, the relatively small number of specimens included for *T. ornata* and *T*. cf. *formosa* does not allow an estimation of the intra and interspecific variability, and species delimitation hypotheses would be more accurately tested by adding replicates. The type locality for *T. formosa* is Panama [29]. The shell of the *T*. cf. *formosa* specimen used in this study (Figure 1B) is very worn and therefore not readily identified, but appears to have the three characteristic squarish brown spots on the body whorl, a short columella that is recurved and heavily plicated, and a smooth subsutural band as described in Bratcher & Cernohorsky [29]. Therefore, as a test of the shell-based ID, the resulting relationships for *T. argyosia, T*. cf. *formosa*, and *T. ornata* are in agreement with what is expected.

### Terebrid toxin characterization

The Panamic *Terebra argyosia/ornata/formosa* complex used in this study have the traits necessary for probing the biochemical characterization of their venom, namely they are found in large quantities and can be easily collected. A combined phylogenetic and toxinological approach will accelerate the investigation of the Terebridae significantly. Genes that encode venom peptides belong to a special category termed “exogenes,” as their gene products act outside the organism [5], [6], [30]. Such genes are expected to diverge from each other extremely rapidly. If the various Panamic forms in the *Terebra* clade are separate species, then their exogenes should have diverged and an entirely different spectrum of venom components would be found in each species. If, however, these are morphological variants of the same species, the same gene sequences (with minor allelic variation) should be observed. Correlating molecular phylogeny with the presence of venom apparatus is a significant advance that will aid in the efficient discovery of new pharmacologically-active compounds from the Terebridae, and also inform the taxonomy and phylogeny of this group.

## References

[1] Puillandre N, Samadi S, Boisselier MC, Sysoev AV, Kantor YI, Cruaud C, Couloux A, Bouchet P (2008). Starting to unravel the toxoglossan knot: molecular phylogeny of the “turrids” (Neogastropoda Conoidea).. Mol Phy & Evol.

[2] Kohn AJ (1959). The Ecology of Conus in Hawaii.. Ecology Monograph.

[3] Taylor JD, Kantor Y, Sysoev AV (1993). Foregut anatomy, feeding mechanisms, relationships and classifications of the Conoidea ( = Toxoglossa) (Gastropoda).. Bull Nat Hist Mus Lond (Zool).

[4] Terlau H, Olivera BM (2004). Conus venoms: a rich source of novel ion channel-targeted peptides.. Physiol Rev.

[5] Olivera BM (2006). Conus peptides: biodiversity-based discovery and exogenomics.. Journal of Biological Chemistry.

[6] Olivera BM, Teichert RW (2007). Diversity of the Neurotoxic Conus peptides: A Model for Concerted Pharmacological Discovery.. Molecular Interventions.

[7] Han T, Teichert RW, Olivera BM, Bulaj G (2008). Conus venoms- A Rich Source of Peptide-Based Therapeutics.. Current Pharmacuetical Design.

[8] Bulaj G (2008). Integrating the discovery pipeline for novel compounds targeting ion channels.. Current Opinion in Chemical Biology.

[9] McIntosh JM, Cruz LJ, Hunkapiller MW, Gray WR, Olivera BM (1982). Isolation and structure of a peptide toxin from the marine snail Conus magus.. Arch Biochem Biophys.

[10] Miljanich G (2004). Ziconotide: neuronal calcium channel blocker for treating severe chronic pain.. Curr Med Chem.

[11] Imperial JS, Watkins M, Chen P, Hillyard DR, Cruz LJ, Olivera BM (2003). The augertoxins: biochemical characterization of venom components from the toxoglossate gastropod Terebra subulata.. Toxicon.

[12] Imperial J, Kantor Y, Watkins M, Heralde FM, Stevenson B, Chen P, Hansson K, Stenfo J, Ownby JP, Bouchet P, Olivera BM (2007). Venomous Auger Snail Hastula (Impages) hectica (Linnaeus, 1758): Molecular Phylogeny, Foregut Anatomy, and Comparative Toxinology.. J Experimental Zoology.

[13] Miller BA (1970). Studies on the biology of Indo-Pacific Terebra (Ph.D. dissertation).

[14] Miller BA (1970). Feeding mechanisms of the family Terebridae.. Ann Rep Am Mal Union.

[15] Taylor JD (1990). The anatomy of the foregut and relationships in the TEREBRIDAE.. Malacologia.

[16] Holford M, Puillandre N, Terryn Y, Cruaud C, Olivera BM, Bouchet P (2009). Evolution of the Toxoglossa Venom Apparatus as Inferred by Molecular Phylogeny of the Terebridae.. Molecular Biology and Evolution.

[17] Terryn Y, Holford M (2008). The Terebridae of the Vanuatu Archipelago with a Revision of the Genus Granuliterebra Oyama 1961..

[18] Keen AM (1972). Sea Shells of Tropical West America. Marine Mollusks from Baja California to Peru. Second Edition..

[19] Simon C, Franke A, Martin A, Hewitt, Johnson AWB, Young JPW (1991). The Polymerase Chain Reaction: DNA Extraction and Amplification, in Molecular Techniques in Taxonomy, G..

[20] Palumbi S, Hillis, Moritz C, Mable BK (1996). Nucleic Acids II: The Polymerase Chain Reaction, in Molecular Systematics, D..

[21] Folmer O, Black M, Hoeh W, Lutz R, Vrijenhoek R (1994). DNA primers for amplification of mitochondrial cytochrome c oxidase subunit I from diverse metazoan invertebrates.. Mol Mar Biol Biotechnol.

[22] Hall T (1999). BioEdit: a user-friendly biological sequence alignment editor and analysis program for Windows 95/98/NT.. Nucl Acids Symp Ser.

[23] Guindon S, Gascuel O (2003). A simple, fast and accurate algorithm to estimate large phylogenies by maximum likelihood.. Syst Biol.

[24] Huelsenbeck JP, Ronquist F, Hall B (2001). MRBAYES: Bayesian inference of phylogeny.. Bioinformatics.

[25] Miller BA (1975). The Biology of Terebra gouldi Deshayes, 1859, and a Discussion of Life History Similarities among Other Terebrids of Similar Proboscis Type.. Pacific Science.

[26] Remigio E, Duda TF (2008). Evolution of ecological specialization and venom of predatory marine gastropod.. Molecular Ecology.

[27] Holford M, Zhang MM, Gowd KH, Azam L, Green BR, Watkins M, Ownby JP, Bulaj G, Olivera BM (2009). Pruning Nature: Biodiversity-Derived Discovery of Novel Sodium Channel Blocking Conotoxins from Conus bullatus.. Toxicon.

[28] McIntosh JM, Corpuz GP, Layer RT, Garrett JE, Wagstaff JD, Bulaj G, Vyazovkina A, Yoshikami D, Cruz LJ, Olivera BM (2000). Isolation and characerization of a novel Conus peptide with apparent antinociceptive activity.. J Biol Chem.

[29] Bratcher T, Cernohorsky WO (1987). Living Terebras of the World..

[30] Imperial J, Silverton N, Olivera BM, Bandyopadhyay PK, Sporning A, Ferber M, Terlau H (2007). Using Chemistry to Reconstruct Evolution: On the Origins of Fish-hunting in Venomous Cone Snails.. Pro Amer Phil Soc.

